# Synthesis of nonylphenol polyoxyethylene oligomer and application as an effective dispersant in pyraclostrobin suspension concentrate

**DOI:** 10.1080/15685551.2019.1616374

**Published:** 2019-05-17

**Authors:** Lei Zhang, Xu Guo, Bo Zhang, Tianrui Ren

**Affiliations:** a The Key Laboratory of Resource Chemistry of Ministry of Education, College of Chemistry and Materials Science, Shanghai Normal University, Shanghai, P. R. China; b State Key Laboratory Breeding Base of Green Pesticide and Agricultural Bioengineering/Key Laboratory of Green Pesticide and Agricultural Bioengineering, Ministry of Education, Guizhou University, Guiyang, P. R. China

**Keywords:** Polyethylene glycol nonylphenyl ether, dispersant, pesticide suspension concentrate, rheology

## Abstract

A versatile dispersant plays a critical role in suspension stability of pesticide aqueous suspension concentrate (SC), thus it is extremely important to design and synthesize an effective dispersant for SC. Herein, nonionic trimeric nonylphenol polyoxyethylene (10) (TNP10) was successfully synthesized using nonylphenol polyoxyethylene(10) ethers (NP10) as raw materials. The surface activities of TNP10 were evaluated according to critical micelle concentration (CMC), and the surface tension at CMC (γ_CMC_). TNP10 showed higher surface activity due to its lower CMC value compared with that of NP10. In addition, the dispersing and stabilizing properties of TNP10 in 25 wt.% pyraclostrobin SC system were investigated. The results showed that TNP10 as a dispersant exhibited more excellent dispersion performance than NP10. Meanwhile, the obtained SC system exhibits shear thinning behavior under high-speed shearing showing typical features of pseudo-plastic non-Newtonian fluids, which conforms to the Herschel-Buckley model. The nonionic trimeric nonylphenol polyoxyethylene (10 can effectively improve the surface activities and the suspension rate and stability of SC compared with its monomer NP10.

## 1. Introduction

Trimeric surfactants, which have three hydrophobic groups, three hydrophilic groups, and a rigid or flexible spacer in the molecule, have been synthesized in a great variety of chemical structures for different applications [1–3]. They exhibited much lower CMC, Kraftt point and better solubility in water than dimeric and monomeric surfactants [3–5]. Different types of trimeric surfactants with varying properties have been synthesized and investigated, including cationic [6,7], anionic [8,9], and nonionic trimeric surfactants [10,11], among which the nonionic trimeric surfactants have been aroused attention increasingly.

A large number of trimeric nonionic surfactants were employed to various applications [12–14]. In addition, they are highly efficient in lowering interfacial tension and form micelles at very low critical micellar concentrations [15,16]. Based on unique superior performance, they have a great potential of being used as effective emulsifiers, bactericidal agents, dispersants, anti-foaming agents, and detergents [17–20]. Trimeric nonionic surfactants are also used in the oil industry recently, and the most of the work is focused on enhanced oil recovery. However, trimeric nonionic surfactants was seldom selected as dispersants in pesticide aqueous suspension concentrate (SC).

SC belongs to a thermodynamic and unstable dispersed system, and easily forms aggregate structures, which enclosing lots of water and result in increasing the viscosity and decreasing the fluidity of SC. Since dispersants can absorb on pesticide particles to effectively improve the stability and rheological properties of SC by electrostatic interaction and/or steric hindrance [21,22].

In this work, we successfully synthesized nonionic trimeric nonylphenol polyoxyethylene (TNP10) using NP10 as monomer. The surface activity properties of TNP10 were determined. Pyraclostrobin is a broad-spectrum, high efficiency, and low-toxicity strobilurin fungicide. TNP10 was selected as a dispersant to prepare 25 wt.% pyraclostrobin SC to verify the suspension stability of SC. Moreover, we further explore the SC stability mechanism using TNP10 as dispersant.

## 2. Experimental

### 2.1. Materials

Nonylphenol polyoxyethylene(10) ethers (NP10), formaldehyde (37%), oxalic acid, ethyl acetate and ethylene glycol were obtained from Sinopharm Chemical Regent Co. (China). Double distilled water was used in the experiment. All chemicals were analytic grade and were used without further purification.

### 2.2. Synthesis of trimer nonylphenol polyoxyethylene ether (TNP10) [23]

**Figure 1.**
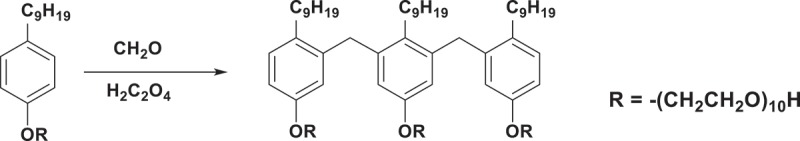
Synthesis route of trimeric nonylphenol polyoxyethylene ether (TNP10).

The synthesis route of trimeric nonylphenol polyoxyethylene ether (TNP10) is shown in Figure 1. Typically, 37% formaldehyde (12 g) was added dropwise to a mixture of NP10 (49 g) and oxalic acid (1 g) at 60 °C within 3 h. The reactants were stirred for 5 h at 95 °C. Then, the water was removed by distillation in vacuum. The crude compound was purified by column chromatography on silica gel (petroleum ether: EtOAc = 1:1) to obtain TNP10 in 85 % yield (41.7 g, 85%). ^1^H NMR (CDCl_3_, 400 MHz), δ: 7.19–7.10 (m, 4H, ArH), 7.05–6.80 (m, 4H, ArH), 4.75 (m, 6H, CH_2_), 4.11 (m, 6H, CH_2_), 3.98 (s, 4H, CH_2_), 3.75–3.10 (m, 108H, CH_2_), 1.71–0.48 (m, 57H, C_9_H_19_); FTIR (cm^−1^) 3485 cm^−1^ (-OH), 2926 and 2871 cm^−1^ (saturated C-H), 1706 cm^−1^ (C = O), 1611, 1517, 1454 cm^−1^ (Ar), 1242, 1161 cm^−1^ (C-O-C), 943, 824 cm^−1^ (Ar). Anal. Calcd for C_107_H_189_O_33_: C, 64.17; H, 9.44; O, 26.39. Found: C, 64.13; H, 9.46; O, 26.41.

### 2.3. CMC values determined by the Wilhelmy plate tensiometry method

Surface tensions of the aqueous solutions were measured at room temperature (25 ℃) with a JYW-2008 tensiometer (CDTM Ltd., China) by Wilhelmy plate method [24]. Prior to each experiment, the instrument was calibrated and checked by measuring the surface tension of distilled water. Stock solutions of TNP10 and NP10 were made in twice distilled water. The CMC value was obtained from the breakpoint of plot of surface tension (*γ*) versus ln *c* [25].

### 2.4. Preparation of 25% pyraclostrobin SC

Pyraclostrobin SC was prepared by wet milling with grinding technology. The optimum formulation of SC was determined by pre-experiment. Typically, 25% pyraclostrobin, 6.0% TNP10, 0.2% xanthan gum, 4% ethylene glycol, 0.1% silicone defoamer were mixed together with the rest of the water, and were ground by using a sand mill to obtain target particle size lower than 5 μm. At the same time, a blank sample was also prepared in which water was used to replace the wetting dispersant. The thermal storage stability and the suspensibility of SCs were determined according to GB/T19136-2003, GB/T19137-2003 and GB/T14825-2006, respectively [26].

### 2.5. Zeta potential of pyraclostrobin SC

1g of pyraclostrobin SC was weighed in a 250 mL beaker using an analytical balance. After diluting the pyraclostrobin to a given concentration by adding deionized water, the solution was transferred to a 50 mL centrifuge tube and centrifuged at 3000 r/min for 5 min. The Zeta potential of the supernatant was measured on a Malvern Nano ZS90. Each sample was measured three times, and the average was used.

### 2.6. Rheological measurement of pyraclostrobin SC

The viscosity and rheological measurements were performed at Anton Par MCR 102 rotational rheometer. The shear viscosity and shear force of the samples were measured at 25°C with the shear rate ranging from 0 to 1000 s^−1^.

Viscosity recovery is an essential property of suspension concentrate, which was measured in three-stages: (i) low shear rate 0.25 s^−1^ for 25 s, (ii) high shear rate of 1000 s^−1^ for 25 s and (iii) low shear rate of 0.25 s^−1^ for 10 s.

## 3. Results and discussion

### 3.1. Critical micelle concentration and surface activity properties

**Figure 2.**
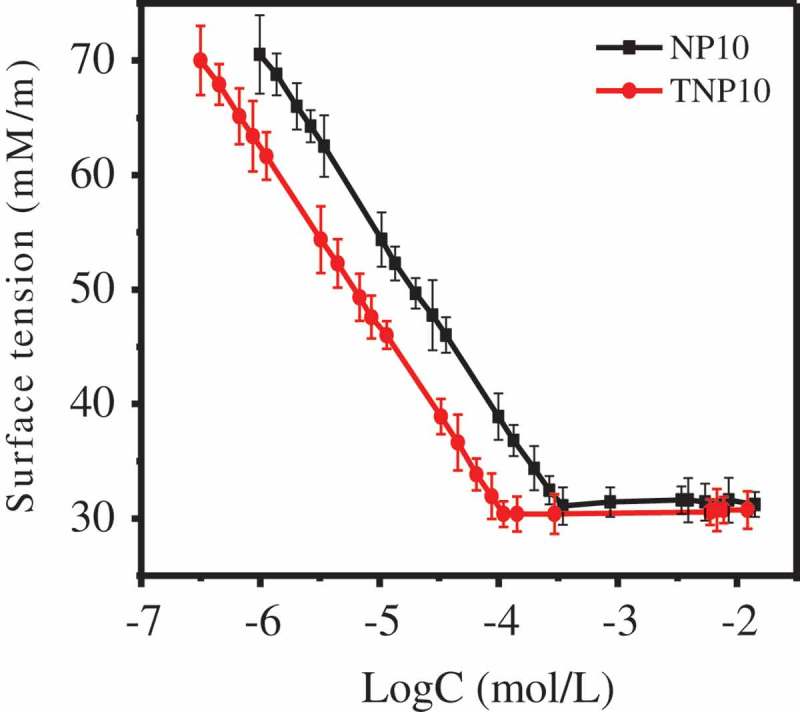
Evaluation of the CMC values of NP10 and TNP10 by means of Wilhelmy plate method.

CMC and surface tension at this concentration (γ_CMC_) are two important indicators to measure the surface activity of surfactant aqueous solution. Figure 2 and Table 1 show the surface tension of NP10 and TNP10, from which we realize that there is a strong decline to decrease the water surface tension. γ_CMC_ of the two surfactants is basically consistent, while CMC of TNP10 is lower than that of NP10 (Table 1), suggesting that the trimeric TNP10 surfactant performs higher surface activity compared with that of NP surfactant.

**Table 1.** Surface properties of NP10 and TNP10.

	γ_CMC_ (mN/m)	cmc (mol/L)	Γ_CMC_ (mol/cm^2^)	A_CMC_ (nm^2^ per molecule)	∆G^θ^_mic_ (kJ/mol)	∆G^θ^_ad_ (kJ/mol)
NP10	31.33	7.8 × 10^−5^	2.65 × 10^−6^	0.6268	−23.45	−38.80
TNP10	30.63	3.3 × 10^−5^	2.0 × 10^−6^	0.8352	−25.58	−46.39

The other surface properties of NP-10 and TNP-10 are also listed in Table 1, including the maximum surface excess concentration, *Γ*_CMC_, the minimum surface area per surfactant molecule, *A*_CMC_, Gibbs free energy of micellisation, ∆*G^θ^_mic_*, and the Gibbs free energy of adsorption ∆*G^θ^_ad_*. They could be calculated by the following equations, respectively [27].

(1) $$\Gamma_{CMC}=\frac{-1}{2.303nTR}(\frac{\partial \gamma }{\partial \mathrm{lg}C}{)}_{T}$$

(2) $$A_{cmc}=\frac{1}{N_{A}\Gamma_{cmc}}$$

(3) $$\Delta G_{mic}^{\Theta }=nRT\ln CMC$$

(4) $$\Delta G_{ad}^{\Theta }=nRT\ln CMC-0.6023(\gamma_{0}-\gamma_{cmc})A_{cmc}$$

in which *γ* represents the surface tension in mN m^−1^, R is the gas constant (8.314 J mol^−1^ K^−1^), T is the absolute temperature, C is the surfactant concentration, and (d*γ*/dlog*C*) is the slope below the CMC in the surface tension plots. *N*_A_ is Avogadro’s number. *γ*_CMC_ represent surface tension at the CMC. *γ*_0_ is the surface tension of pure water. The value of n is the number of ionic species whose concentration at the interface varies with the surfactant concentration in the solution (n was 1 for non-ionic surfactants).

It is noteworthy that the area per surfactant molecule (A_CMC_) of TNP10 is three times smaller than that of NP10, suggesting that the trimeric surfactant molecules of TNP10 are not arranged side by side at the air-water interface, but staggered three-demensional arrangement [28,29]. Compared with monomer NP10, TNP10 has lower CMC and smaller A_CMC_. Hence, TNP surfactants exhibit much better surface activities, including strong molecule adsorption and wetting ability at the surface.

In addition, the values of ΔG^Θ^_mic_ and ΔG^Θ^_ad_ were negative, which indicated that two surfactants can spontaneously self-assemble into micelles in solution, and effectively adsorbed at the air-water interface [30]. Meanwhile, in comparison with NP10, TNP10 has a lower Gibbs free energy (−25.58 kJ/mol), suggesting that TNP10 has a greater tendency to form micelles. Furthermore, the absolute value of ΔG^Θ^_ad_ of TNP10 was significantly greater than that of NP10, suggesting that in comparison to micellisation, the adsorption of TNP10 is more favourable [27].

### 3.2. Effect of dispersant on Zeta potential and viscosity of pyraclostrobin SC

**Figure 3.**
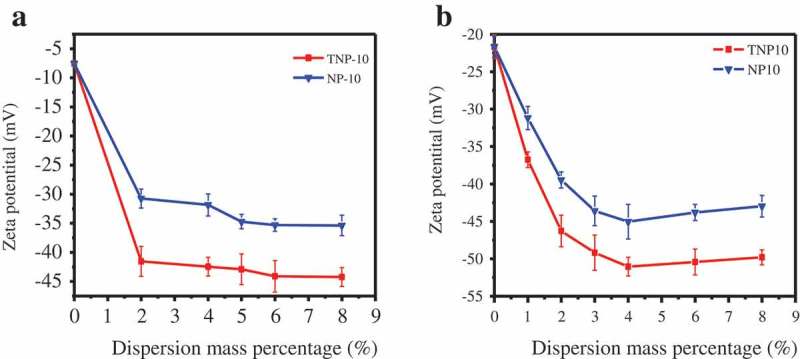
Impacts of NP10 and TNP10 on zeta potential of pyraclostrobin SC before (a) and after (b) thermal storage, respectively.

The dispersive ability of TNP10 and NP10 was determined by the adsorption on pyraclostrobin particles. Effective adsorption of dispersants on particles is crucial to improve the stability of SC, which can significantly influence on zeta potential and viscosity of suspension systems by adsorbing on the particles surface to form steric effect and electrostatic repulsion among particles and keep suspensions stability. Figure 3(a,b) depicted the effects of NP10 and TNP10 on the Zeta potential of pyraclostrobin SC before and after thermal storage, respectively. It was found that the amount of dispersant dramatically affected the zeta potential, and the absolute values of zeta potentials of TNP on particle surface are lower than those of NP. The zeta potential decreases sharply with increasing dispersant from 0 to 2 wt.%, and decrease slightly when exceed 2%. With increasing amount of dispersants, the absolute values of potentials on particle surface keep increasing. Furthermore, at the concentration of 6%, the zeta potential absolute value of pyraclostrobin reached the maximum of NP10 and TNP10. This behaviour is due to its maximum adsorption on the pyraclostrobin surface [31].

Surprisingly, when the amount of dispersant was more than 6 wt.%, the zeta potential absolute value of pyraclostrobin decreased. This may be that the amount of dispersants exceeds the saturated adsorption on the pesticide surface, excessive counter ions in dispersants enter into the diffusion layer in the double electrical layer, resulting in the slight decrease in Zeta potential values. In addition, Figure 3(a,b) also reveals that thermal storage has a minor effect on zeta potentials of SCs, suggesting the dispersants can dramatically improve the SCs stability performance.

**Figure 4.**
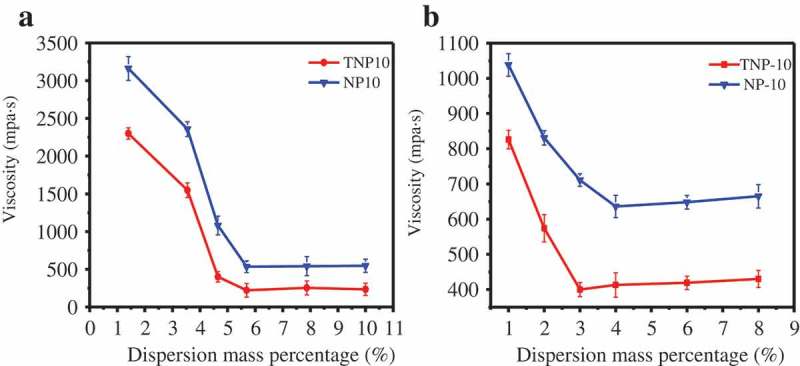
Effect of dispersant amount on the viscosity of pyraclostrobin SC before (a) and after (b) thermal storage, respectively.

The dispersant can effectively improve the system viscosity, and further effect on suspension stability. Figure 4 depicted the influence of dispersant amount on viscosity of pyraclostrobin SC. As shown in Figure 4, when the amount of the dispersants varied from 1 wt.% to 6 wt.%, the viscosity of pyraclostrobin SC before and after thermal storage sharply decreased. More specifically, when the dispersant dosage is 6 wt.%, the viscosity of the pyraclostrobin SC is the lowest, which agrees with the zeta potential (Figure 3). In addition, when the amounts of dispersant are less than 6 wt.%, the pyraclostrobin particle surface cannot be saturated with the dispersant, and easily form agglomerate, which leads to the viscosity increase of SCs systems [32,33]. However, the further increasing the amount of dispersants increased the system viscosity, which is because that the excess dispersant causes counterions to enter the diffusion layer and the electrostatic repulsion decreases. Thus, the combined bridging and depletion effects result in increased viscosity of the suspension system [34].

Notably, compared with NP10, TNP10 can more effectively improve the viscosity of pyraclostrobin SC system. A possible explanation is that TNP10 has strong molecular adsorption and staggered three-dimensional arrangement on the particle surface (Table 1), which arouse larger steric hindrance than NP10 to favour the stability SCs systems.

Additionally, thermal stability and rheological properties of pyraclostrobin SC made by TNP10 in 6 wt.% dosage were further investigated.

### 3.3. Thermal stability of pyraclostrobin SC

**Figure 5.**
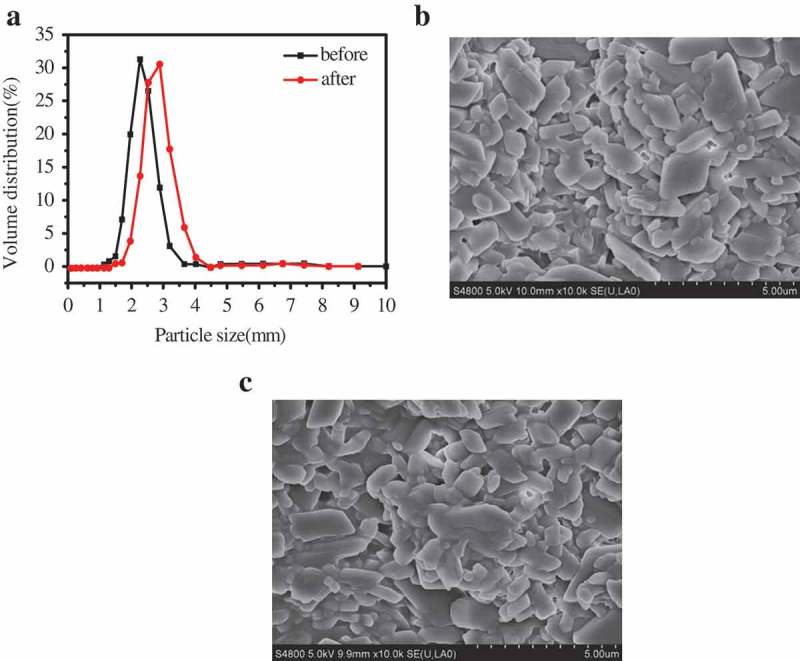
Particle size distributions (a) and FESEM images (b and c) of pyraclostrobin SC prepared by 6 wt.% TNP10 before and after thermal storage, respectively.

The particle size distribution has a significant impact on system viscosity and suspension stability. Figure 5(a) depicts the particle size distribution of the pyraclostrobin SC prepared by 6 wt.% TNP10 before and after thermal storage at 54 ± 2°C for 14 d. The average diameter of particles before and after thermal storage is about 3.226 and 3.78 μm, respectively, indicating TNP10 can effectively inhibit crystal growth in thermal storage process, which is consistent with the results of FESEM images of pyraclostrobin particle (Figure 5(b,c)). From the FESEM images (Figure 5(b,c)), it clearly depicts that pyraclostrobin particles have no obvious aggregation before and after thermal storage.

### 3.4. Analysis of rheological properties of pyraclostrobin SC

**Figure 6.**
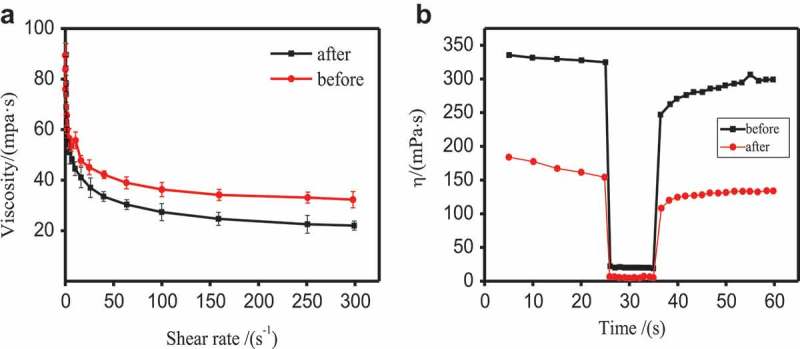
Rheological properties (a) and three-stage shearing curve (b) of pyraclostrobin SC prepared by 6 wt.% TNP10 before and after thermal storage, respectively.

Pyraclostrobin SC prepared by 6 wt.% TNP10 showed excellent dispersion performance. The suspension stability of SC system were also investigated by rheological method. Apparent viscosity and thixotropy can especially reflect the suspension stability of SC [35,36]. Figure 6(a) shows the rheological curves of the 25 wt.% pyraclostrobin SC used TNP10 as dispersant before and after thermal storage at 54 ± 2 °C. As Figure 6(a) shows, the apparent viscosity of pyraclostrobin SC decreases by increasing shear rate until a steady state was approached and showed a typical shear thinning behaviour, which is in accord with typical non-Newtonian pseudo-plastic fluid. In other words, with the increase of shear rate, the directions and arrangements of the particles in the suspension system changed, and the network structure was destroyed, leading to decline of apparent viscosity. Moreover, when the shear rate is greater than 20 s^−1^ (Figure 6(b)), the network structure of the system is completely destructed, then the apparent viscosity remains unchanged, and the system shows the characteristic of shear thinning [37].

The three-stage thixotropy and modulus-strain tests were used to verify the viscosity recovery of pyraclostrobin SC, and can reflect the relationship between the viscosity and the change of network structure in the pyraclostrobin SC system. Figure 6(b) and Table 2 show the results of 3-step thixotropy test. The test results indicated when at low-speed shear (0.25 s^−1^ shear rate before 25s), the spatial network structure of the SC system appear stable, and the system has high viscosity. Whereas when the shear rate is increased to 1000 s^−1^, the structure of the system was partly damaged, resulting in a rapid decline in viscosity. In the third stage (0.25 s^−1^ after 35s), the structure of SC system was restored, and the viscosity increased. The recovery rate is greater than 80% at 15 s both before and after high-temperature storage. The rapid recovery of the system structure can prevent the separation of solid-liquid phases from destroying the suspension stability.

**Table 2.** Rheological analysis of three-stage shearing of 25 wt.% pyraclostrobin SC made by TNP10.

Sample	Structure recovery ratio (%)			
	5 s	7 s	10 s	15 s
Before thermal storage	80.99	82.98	84.63	86.32
After thermal storage	71.70	78.12	80.3	81.22

### 3.5. Proposed stability mechanism of pyraclostrobin SC prepared by TNP10

**Figure 7.**
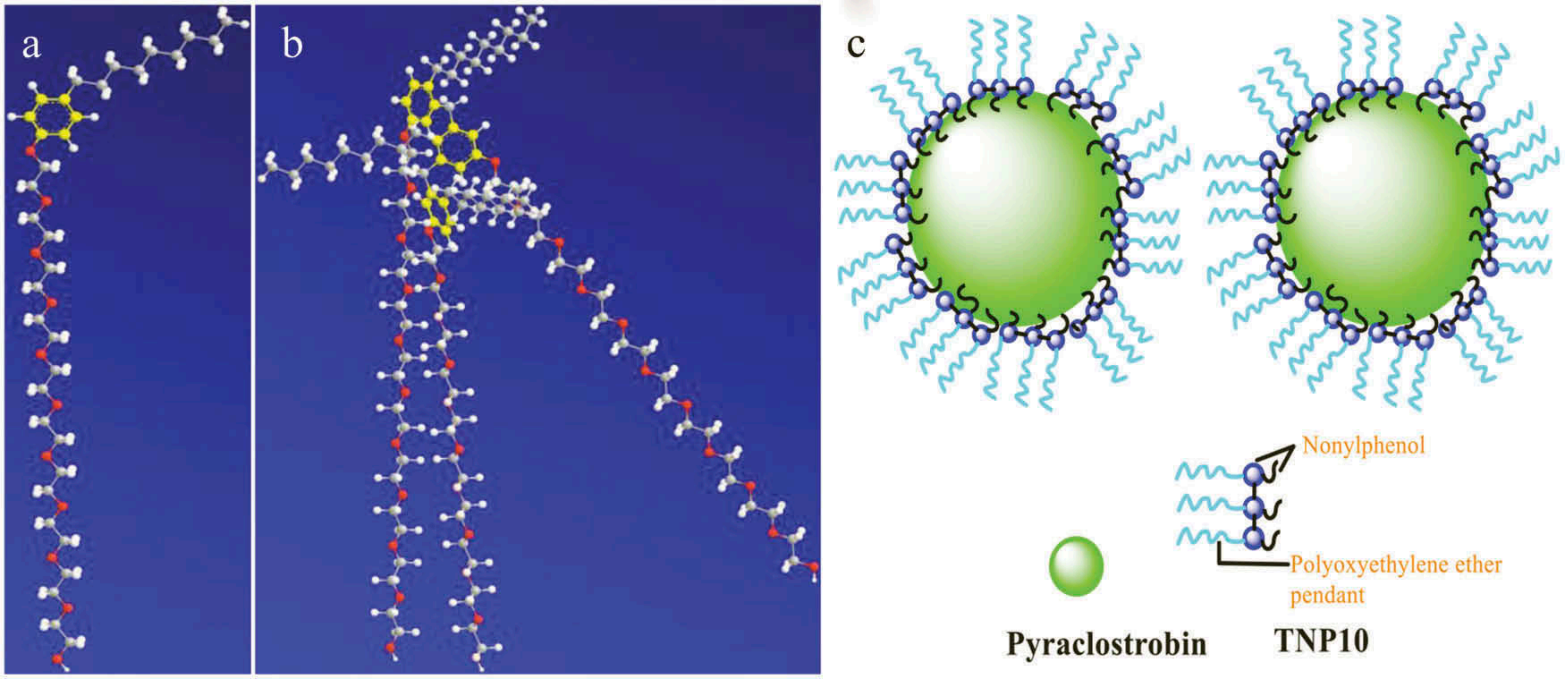
Conformations of (a) NP10 and (b) TNP10 as well as (c) Dispersing and stabilizing mechanism of TNP10 dispersants in pyraclostrobin SC system.

In the process of pesticide particles dispersing into aqueous solution, the pesticide particles were firstly wet by aqueous solutions, and by increasing the wettability of the pesticide surfaces, dispersants reduce the frequently observed tendency of hydrophobic pesticide particles to spontaneously aggregate in aqueous suspensions, therefore, the lower surface tension of the aqueous solution is, the easier the wetting of pesticide particles so as to help the pesticide particles more readily disperse into aqueous solution [38]. Compared with monomer NP10, TNP10 has lower CMC (Table 1). Hence, TNP10 exhibit much better strong molecule adsorption and wetting ability at the surface, and are more conducive to wet the pesticide surface. Furthermore, to clarify the potential mechanism of dispersing behaviour of NP10 and TNP10 dispersant, we provided the possible stereo structure of NP10 and TNP10 simulated by ChemBiooffice 3D software and the result is shown in Figure 7(a,b). As Figure 7(b) shows that three terminal groups of TNP10 are chemically bonded through two methylene, which makes three monomer NP10 molecules closely connected, and hydrocarbon chains easy to produce strong interactions. Moreover, TNP10 have three long hydrophilic EO pendants. Therefore, steric hindrance can be obtained when the hydrophilic pendants of adsorbed TNP10 stretch out from the surface of pyraclostrobin particle into water and form a dense layer. Mechanism of dispersion and stability improvement of pyraclostrobin SC by TNP10 dispersant are proposed and illustrated in Figure 7(c).

TNP10 has hydrophobic aromatic and nonyl pendants, which make them steadily be adsorbed on the surface of pyraclostrobin particle. Meanwhile, TNP10 contains neutral EO pendants, which provide steric hindrance among pyraclostrobin particles. For above reasons, TNP10 is endowed with an excellent ability to stabilize and disperse pyraclostrobin particles, and maintained the suspension stability of pyraclostrobin SC.

## 4. Conclusion

Trimeric TNP10 was successfully synthesised using NP10 as monomer. The surface activity properties experimental results show that the CMC value of TNP10 is lower than that of the corresponding monomeric NP10. TNP10 is very more favourable for the formation of micelles compared with NP10. Moreover, the area per surfactant molecule of TNP10 is three times smaller than that of NP10, which indicates that the TNP10 are staggered three-dimensional arrangement, not arranged side by side at air-water interface. More significantly, TNP10 can serve as a suitable dispersant to stabilize the 25 wt.% pyraclostrobin SC. When its dosage was 6 wt.%, the pyraclostrobin SC had the minimum viscosity and the maximum zeta potential, which is benefit to keeping the suspension stability of SC system. Moreover, its rheological properties was further evidence that the 25 wt.% pyraclostrobin SC using TNP10 as dispersant exhibit excellent suspension stability compared to NP10.
